# Associations of serotonin transporter gene promoter polymorphisms and monoamine oxidase A gene polymorphisms with oppositional defiant disorder in a Chinese Han population

**DOI:** 10.1186/s12993-018-0147-6

**Published:** 2018-08-20

**Authors:** Chang-Hong Wang, Qiu-Fen Ning, Cong Liu, Ting-Ting Lv, En-Zhao Cong, Jing-Yang Gu, Ying-Li Zhang, Hui-Yao Nie, Xiao-Li Zhang, Yan Li, Xiang-Yang Zhang, Lin-Yan Su

**Affiliations:** 1 Department of Psychiatry, The Second Affiliated Hospital of Xinxiang Medical University (Psychiatric Hospital of Henan Province China), Jianshe Road 388, Xinxiang, 453002 Henan China; 2 Department of Child and Adolescent, Public Health College, Zhengzhou University, Kexue Road 100, Zhengzhou, 450001 Henan China; 3 Department of Psychiatry and Behavioral Sciences, UT Houston Medical School, The University of Texas Health Science Center at Houston, 1941 East Road, Houston, TX 77054 USA; 4 Department of Psychiatry, Mental Health Institute, Second Xiangya Hospital of Central South University, Changsha, 410011 China

**Keywords:** Oppositional defiant disorder, Serotonin transporter, Monoamine oxidase A, Gene polymorphism

## Abstract

**Background:**

Oppositional defiant disorder (ODD) is a behavioral disorder that mainly refers to a recurrent pattern of disobedient, defiant, negativistic and hostile behaviors toward authority figures. Previous studies have showed associations of serotonin transporter (5-HTT) and monoamine oxidase A (MAOA) with behavioral and psychiatric disorders. The purposes of this study were to investigate the potential association of 5-HTT gene promoter polymorphism (5-HTTLPR) and MAOA gene polymorphism with susceptibility to ODD in a Han Chinese school population.

**Methods:**

The 5-HTTLPR gene polymorphism and the MAOA gene polymorphism were genotyped in a case–control study of 257 Han Chinese children (123 ODD and 134 healthy controls).

**Results:**

There was significant difference in the allele distribution of 5-HTTLPR (χ^2^ = 7.849, *P *= 0.005) between the ODD and control groups. Further, there were significant differences in genotype (χ^2^ = 5.168, *P *= 0.023) and allele distributions (χ^2^ = 10.336, *P *= 0.001) of the MAOA gene polymorphism that is variable-number tandem repeat (MAOA-uVNTR) between two groups. Moreover, there were significant differences in genotype (χ^2^ = 4.624, *P *= 0.032) and allele distributions (χ^2^ = 9.248, *P *= 0.002) of MAOA-uVNTR only in the male ODD and healthy groups.

**Conclusions:**

Our results suggest that 5-HTTLPR and MAOA-uVNTR gene variants may contribute to susceptibility to ODD. Further, MAOA-uVNTR gene polymorphism may play a role in susceptibility to ODD only in male children.

## Background

Oppositional defiant disorder (ODD), a common childhood disorder, is a behavioral disorder that mainly refers to a recurrent pattern of disobedient, defiant, negativistic and hostile behavior toward authority figures [1–3]. Even mild stimulation can lead to impulsive actions or intense emotional reaction from the children with ODD, since they may have trouble in controlling their temper. Accordingly, ODD is also deemed as a disorder of emotional adjustment [4]. ODD can lead to a large number of negative outcomes that are dreadful to their lifetime, such as anxiety, some psychiatric problems, unemployment or even delinquency [5].

Nowadays, ODD is one of the leading reasons for referral to youth mental health services [6]. A survey [7] based on Diagnostic and Statistical Manual of Mental Disorders-IV-Text Revision (DSM-IV-TR) from an Asian country showed that the morbidity for ODD is 2% to 16%, with the ODD prevalence of 3.87% in the Turkish children [8]. As a result, the disorder has now become a hot spot of research in the field of children’s mental health. Unfortunately, the pathological mechanisms of ODD are still unclear yet. The previous studies have indicated that ODD possesses some of its own characteristics such as moderate heritability, substantially stable over time [9], and familial clustering [1], suggesting an underlying genetic component.

Serotonin (5-HT) is one of crucial neurotransmitters that controls the behaviors of the individuals, such as appetite, locomotion, aggression and attention. It has been demonstrated that deficiency in the 5-HT functions has some serious effects on increased pain sensation, anxiety, aggression, attention deficit hyperactivity disorder (ADHD) symptoms [10], and even impulsivity, which are common characteristics of substance abuse [11, 12], ODD and personality disorder [13, 14]. Early studies have reported that impulsive attacking activities, and antisocial behaviors, as well as other “de-inhibition” behaviors, are closely related to dysfunction of the brain 5-HT system [15, 16]. A strong relationship between impulsive aggression and low cerebrospinal fluid 5-HT has been reported in the previous study [17]. It appears that ODD is also associated with dysfunctions of the 5-HT system [13, 14].

Furthermore, the related enzymes, such as monoamine oxidase A (MAOA) can regulate the synthesis, transfer, and decomposition of 5-HT, and may also play an important role in the development of ODD. Accumulating evidence has demonstrated the association between ADHD and 5-HT, 5-HTT or MAOA [18]. Meanwhile, it was reported that the serotonin transporter gene promoter polymorphism (5-HTTLPR) was associated with borderline personality disorder in young people [19], and with antisocial personality disorder [20]. On the other hand, MAOA genotype was found to be associated with impulsive and antisocial personality traits in alcoholic men [21]. MAOA also contributed to the pathogenesis of aggression [22, 23] and antisocial personality [24]. A previous study reported a 30 bp variable number of tandem repeats (VNTR) polymorphism located in the upstream MAOA promoter region (MAOA-uVNTR) [25]. The VNTR alleles consist of 2 repeats, 3 repeats, 3.5 repeats, 4 repeats, or 5 repeats. Alleles with 3.5 or 4 copies of the repeat sequence were associated with higher MAOA gene activity (MAOA-H), while alleles with 2, 3 or 5 copies of the repeat sequence were associated with lower gene activity (MAOA-L), demonstrating that this polymorphism may strongly affect the transcriptional efficiency of MAOA gene, and may be regarded as a functional genetic marker for MAOA gene [23].

In terms of the associations of 5-HTTLPR and MAOA-uVNTR with behavioral and psychiatric disorders in previous studies, it would be of interest to examine the association between 5-HTTLPR or MAOA-uVNTR and ODD, which, to our best knowledge, has not been investigated. Therefore, the present study was designed to investigate the potential association of 5-HTTLPR or MAOA-uVNTR with susceptibility to ODD in a Han Chinese school population.

## Materials and methods

The present study was designed in compliance with the Helsinki Declaration and was approved by the Ethical Committee of the Second Affiliated Hospital, Xinxiang Medical College, Henan Province. Written informed consent was obtained from all subjects and their parents.

## Study participants

We used random group sampling method to collect samples from 2000 Chinese Han pupil in Nanyang City, Henan Province, China, between 2007 and 2009. All their teachers completed Conners Teachers Rating Scale. In order to conform to the diagnostic criteria, a chief physician and resident physician had an interview with one or both parents as well as the teachers, and then made a diagnosis of ODD based on Diagnostic and Statistical Manual of Mental Disorders-IV (DSM-IV) diagnostic criteria. We also recruited the healthy control subjects from the same primary school as control group.

The exclusion criteria included: (1) intelligence quotient (IQ) < 70 (IQ criteria was based on Raven’s Progressive Matrices); (2) low body mass index (BMI, < 18.5 kg/m^2^); and (3) any physical diseases, ADHD or Conduct disorder (CD) symptoms, as well as other mental illnesses.

Totally, we recruited 123 ODD (male/female = 70/53) with an average age of 10.4 ± 1.8 years, as well as 134 healthy controls (male/female = 66/68) with an average age of 10.1 ± 2.0 years. They matched on age (*t *= 1.969, *P *= 0.209) and gender (χ^2^ = 1.509, *P *= 0.219).

## Genotyping

5 ml peripheral blood samples from cubital vein of each subject were drawn into vacutainer tubes containing the anticoagulant ethylenediaminetetracetic acid (EDTA) between 8:00 and 9:00 am after an overnight fasting. Blood samples were stored at − 70 °C until being assayed.

Genomic DNA was isolated from the whole blood using a standard phenol–chloroform technique (the human leukocyte DNA extraction kit from TianGen Biotech), in accordance with the manufacturer’s instructions.

The polymorphism of the 5-HTTLPR was genotyped using a polymerase chain reaction (PCR). Primers for the PCR were designed as described in a previous study [26]. The PCR products were assayed using gel electrophoresis and genotype analysis.

The PCR conditions for polymorphisms of 5-HT gene were as follows: DNA was denatured at 95 °C for 4 min and subjected to 34 cycles of 40 s denaturation at 94 °C, 40 s annealing at 63 °C, 40 s extension at 72 °C, and a final extension step of 72 °C for 10 min. The PCR products were then analyzed on 7% polyacrylamide gels that were stained with ethidium bromide. Product sizes for cleaved products were: L (528 base pairs) and S (484 base pairs).

The MAOA-uVNTR was genotyped as described by Manor et al. [27]. The MAOA-uVNTR genotypes were divided into three groups: 3/3 (low activity allele), 3/4 and 4/4 (high activity alleles) [28]. Primers for the PCR were designed as described in a previous study [28]. The PCR conditions for MAOA gene were as follows: DNA was denatured at 95 °C for 4 min and subjected to 34 cycles of 40 s denaturation at 94 °C, 40 s annealing at 54 °C, 40 s extension at 72 °C, and a final extension step of 72 °C for 10 min.

## Statistical analysis

PASW Statistics 13.0 software (SPSS Inc., Chicago, IL, USA) was used to analyze the differences in allele and genotype frequencies between the two groups, with Chi squared test for categorical variables.

We used Chi square (χ^2^) goodness-of-fit test to test the deviation from the Hardy–Weinberg equilibrium (HWE) in cases and controls separately according to previous study [29].

The level of significance was set at *P *= 0.025 after Bonferroni correction.

## Results

### Hardy–Weinberg genetic equilibrium testing

The genotypic distributions of the 5-HTTLPR were in agreement with the HWE in both the ODD (χ^2^ = 1.3831, df = 1, *P *> 0.05) and control groups (χ^2^ = 1.4758, df = 1, *P *> 0.05). The genotypic distributions of MAOA-uVNTR were in agreement with the HWE in females, in both the ODD (χ^2^ = 0.422, df = 1, *P *= 0.516) and control groups (χ^2^ = 0.107, df = 1, *P *= 0.743). The genotype and allele frequencies of HTTLPR and MAOA-uTNTR in the ODD and control groups are shown in Tables 1 and 2.

**Table 1:** Distribution of polymorphisms of the 5-HTTLPR gene in the ODD group and controls

	Genotype (%)			χ^2^	*P*	Alleles (%)		χ^2^	*P*
	LL	LS	SS			L	S		
ODD (n = 123)	7 (5.7)	10 (8.1)	106 (86.2)	5.559	0.062	24 (9.8)	222 (90.2)	7.849	0.005*
Controls (n = 133)	15 (11.3)	19 (14.3)	99 (74.4)			49 (18.4)	217 (81.6)		
OR (95% CI)	0.44 (0.17–1.11)	0.49 (0.22–1.11)	1.00			0.48 (0.28–0.80)	2.09 (1.24–3.52)		

df = 1, *P < 0.05

**Table 2:** Distribution of polymorphisms of the MAO-A gene in the ODD group and controls

Genotype	3/3 (%)	3/4 and 4/4 (%)	χ^2^	*P*
Male				
ODD (*n *= 70)	13 (18.6)	57 (81.4)	4.624	0.032*
Controls (*n *= 66)	23 (34.8)	43 (65.2)		
*OR(95%CI)*	0.43 (0.94–0.19)	2.35 (1.07–5.15)		
Female				
ODD (*n *= 53)	5 (9.4%)	48 (90.6%)	1.664	0.197
Controls (n = 68)	12 (17.6%)	56 (82.4%)		
*OR(95%CI)*	0.49 (0.16–1.48)	2.06 (0.68–6.26)		
Alleles				
Female				
ODD (*n *= 53)	10 (9.4%)	96 (90.6%)	3.328	0.068
Controls (*n *= 68)	24 (17.6%)	112 (82.4%)		

df = 1, *P < 0.05

### Comparison of 5-HTTLPR genotype and allele frequencies between ODD and control groups

Although there were no significant differences in the 5-HTTLPR genotype distributions between the ODD and control groups (χ^2^ = 5.559, *P *= 0.062), there were statistically significant differences in the allele distributions (χ^2^ = 7.849, *P *= 0.005). With the dominant model (SS VS LL-LS), the genotype distributions displayed significant differences between the case and control groups (*χ*^*2*^= 5.559, *P *= 0.062; OR = 0.58, 95% CI 0.44 − 0.78).

### Comparison of MAOA-uVNTR genotype and allele frequencies between ODD and control groups

There were significant differences in the genotype (χ^2^ = 5.168, *P *= 0.023) and allele distributions (χ^2^ = 10.336, *P *= 0.001) of MAOA-uVNTR between the ODD and control groups. Further, there were also significant differences in the allele (χ^2^ = 9.248, *P *= 0.002) and genotype (χ^2^ = 4.624, *P *= 0.032) distributions between the male ODD and male control groups, but no significant differences in genotype (χ^2^ = 1.664, *P *= 0.197) and allele distributions (χ^2^ = 3.328, *P *= 0.068) between the female ODD and female control groups.

## Discussion

To our knowledge, this is the first study to confirm an association between the 5-HTTLPR or MAOA-uVNTR polymorphism and ODD in a Chinese Han population.

Although no significant differences were observed in the 5-HTTLPR genotype frequencies between the ODD and control groups, the allele frequencies of the two groups showed significant differences. The significantly lower L allele frequency, but the significantly higher S allele frequency was noted in the ODD children compared with the control children. Thus, the S allele of 5-HTTLPR may increase the risk for ODD, whilst the L allele may be protective. These results are consistent with previous reports showing that the S allele was associated with violent suicide [30] and violence, ADHD [31], PTSD, alcohol dependence, and conduct problems [32], but not with personality traits [33]. However, a study showed that the L allele of 5-HTTLPR might increase the risk of attacking behaviors in the Alzheimer’s patients [34], which is contrary to our findings. The reasons for the discrepancies between these 2 studies are unclear, but may be related to different study subjects, i.e., senior patients in the Alzheimer’s study versus children in our ODD study.

The MAOA-uVNTR genotype and allele distributions were significantly different between the ODD and control groups. Further analysis showed that the high-activity allele of MAOA-uVNTR may increase the risk for ODD in children, whilst the low-activity allele may be a protective factor, which are consistent with previous studies showing that MAOA-uVNTR was associated with impulsive behaviors and alcohol dependence [35], aggressive behaviors [36, 37] and suicide attempts in patients with major depressive disorder [38]. In contrast, some studies [39–41] indicated that boys suffering from abuse with the low-activity allele of MAOA-uVNTR were at higher risk of developing antisocial behaviors. Another study found that aggressive behaviors were also associated with the low-activity allele of MAOA-uVNTR [42]. Additionally, some reports showed no correlation between MAOA-uVNTR and impulsive behavior [43, 44]. The reasons for the discrepancies between these studies may be related to either the different methods adopted by different researchers, or the difference in the number of study samples.

Violence is thought to be a male trait, which may be controlled by a gene on the Y chromosome. However, the gene that controls the trait and produces MAOA can only be found on the X chromosome [25]. There are two X chromosomes in females, and only one X chromosome and one Y chromosome in males. To determine whether sex influenced the MAOA-uVNTR results, we specifically performed further statistical analysis on the male ODD versus male control groups or on the female ODD versus control groups separately, showing the genotype and allele distributions of MAOA-uVNTR were significantly different only between the male ODD and control groups. This indicated that MAOA-uVNTR was only associated with male ODD patients. Our results demonstrated that the high-activity allele of MAOA-uVNTR may increase the risk for male ODD. Taken together, these results suggested that MAOA-uVNTR gene polymorphism may be susceptible for ODD in male children, which is consistent with to a previous study showing association between MAOA-VNTR and aggressive behavior in males [45].

The samples in this study were mainly collected from local schools in Nanyang City, Henan Province, China. Thus, the subjects from the single geographical location may have bias for the results. Moreover, there is only the limited sample size in the current study, with low statistical power. Thus, the findings in our present study remain preliminary and future studies with an increased number of samples from the multiple locations will be needed.

## Conclusion

The S allele of 5-HTTLPR may increase the risk for ODD in Chinese Han children, whereas the L allele may be a protective factor. The high-activity allele of MAOA-uVNTR may increase the risk for ODD in Chinese Han male children.

## Abbreviations

ODD: oppositional defiant disorder
5-HTT: serotonin transporter
MAOA: monoamine oxidase A
5-HTTLPR: serotonin transporter gene promoter polymorphism
MAOA-uVNTR: monoamine oxidase A- untranslated variable nucleotide tandem repeat
PCR: polymerase chain reaction
DSM-IV-TR: Diagnostic and Statistical Manual of Mental Disorders-IV-Text Revision
ADHD: attention-deficit/hyperactivity disorder
MAOA-H: higher MAOA gene activity
MAOA-L: lower MAOA gene activity
DSM-IV: Diagnostic and Statistical Manual of Mental Disorders-IV
IQ: intelligence quotient
BMI: body mass index
CD: conduct disorder
EDTA: ethylenediaminetetracetic acid
ANOVA: one-way analysis of variance
HWE: Hardy–Weinberg equilibrium
